# Adherence to a healthy Nordic diet is associated with a lower prevalence of depressive symptoms

**DOI:** 10.1017/S0007114525103772

**Published:** 2025-07-14

**Authors:** Johanna Roponen, Jyrki K. Virtanen, Timo Partonen, Pilvikki Absetz, Sari Hantunen, Tomi-Pekka Tuomainen, Outi Nuutinen, Tommi Tolmunen, Anu Ruusunen

**Affiliations:** 1 Institute of Public Health and Clinical Nutrition, University of Eastern Finland, PL 1627/Yliopistonrinne 3, Canthia, 70211 Kuopio, Finland; 2 Department of Healthcare and Social Welfare, Finnish Institute for Health and Welfare, P.O. Box 30 (Mannerheimintie 166), 00271 Helsinki, Finland; 3 Faculty of Social Sciences, Tampere University, Tampere 22014, Finland; 4 Institute of Clinical Medicine, University of Eastern Finland, Yliopistonranta 1, 70210 Kuopio, Finland; 5 Department of Adolescent Psychiatry, Kuopio University Hospital, Kaartokatu 9, Kuopio, Finland; 6 Mental health and Wellbeing, Kuopio University Hospital, Wellbeing Services County of North Savo, Puijonlaaksontie 2, 70210 Kuopio, Finland; 7 IMPACT – the Institute for Mental and Physical Health and Clinical Translation, Food & Mood Centre, School of Medicine, Faculty of Health, Deakin University, Barwon Health, 18 PO Box 281, Geelong, VIC 3220, Australia

**Keywords:** Depression, Depressive symptoms, Diet, Diet quality, Healthy Nordic diet

## Abstract

There has been substantial research undertaken on the role of a health-promoting diet in depression. Yet, the evidence of the relationship between the Nordic diet and the risk of depression is scarce. This cross-sectional study aimed to assess whether a healthy Nordic diet is associated with depressive symptoms. In total, 2603 men aged 42–60 years from the Kuopio Ischaemic Heart Disease Risk Factor Study were included. Diet quality was evaluated with a healthy Nordic diet score derived from the 4-day food diaries and depressive symptoms with the self-reported Human Population Laboratory (HPL) depression scale. Quade ANCOVA was used to examine the mean values of HPL scores in quartiles of a healthy Nordic diet score. Participants’ mean age was 53 years and BMI 26·8 kg/m^2^; 31·7 % were current smokers, and 86·9 % were married or living as a couple. The mean healthy Nordic diet score was 12·8 (sd 4·0, range 2–25), and the mean HPL depression score was 1·9 (sd 2·1, range 0–13). The findings suggested that lower adherence to a healthy Nordic diet was associated with higher HPL depression scores after adjusting for age, examination year, daily energy intake, leisure-time physical activity, adulthood socio-economic status, smoking and marital status (extreme quartile difference: 0·33 points, 95 % CI 0·10, 0·56, *P* for trend across the quartiles = 0·003). The results support the hypothesis that a lower-quality diet increases the odds of having depressive symptoms. However, prospective studies are needed to confirm the association.

The depressive disorder has become a global health challenge that diminishes both physical and psychological well-being, work ability and quality of life^(1,2)^, which causes substantial economic losses^(3,4)^. The high prevalence of depressive disorder and the challenges related to its treatment^(5,6)^ highlight the need for new preventive approaches. Diet, as a modifiable risk factor, could offer a feasible way to prevent depressive symptoms. Suggested pathways through which dietary intake could influence the odds of depressive symptoms are related to, for example, gut microbiome, inflammation and oxidative stress^(7)^. Recent research has examined how specific nutrients and certain foods are associated with the risk of depressive disorder^(8–19)^. However, instead of focusing on individual foods and nutrients, studying diet quality and dietary patterns represents a broader picture of food consumption and nutrient intake^(20)^. Quality of diet can be assessed with diet quality scores, which can be calculated based on predefined algorithms to quantify food and nutrient intake relative to nutritional recommendations (*a priori* method). In contrast, a *posteriori* assessment does not rely on predefined guidelines but rather derives the dietary patterns from dietary data that emerge naturally in a given population.^(21)^

Currently, the strongest evidence of the inverse association between diet quality and depressive disorder exists for the Mediterranean diet^(22)^. Similar evidence has been found with low adherence to a pro-inflammatory diet^(22)^ and with a healthy dietary pattern evaluated with different (country-specific) dietary quality scores or indices^(22,23)^. Molendijk et al.^(24)^ reported in their systematic review of prospective cohort studies that a higher quality diet, irrespective of the pattern (e.g. healthy/prudent, Mediterranean, pro-vegetarian, Tuscan), was associated with a lower risk for the onset of depressive symptoms. However, no studies conducted with a Nordic diet were included^(24)^.

A typical Nordic diet shares many elements with the Mediterranean diet. Both emphasise plant-based foods, contain moderate amounts of fish, eggs and small amounts of dairy products and limit the consumption of red meat, processed foods and sweets. The Nordic diet favours rapeseed oil instead of olive oil used in the Mediterranean diet. The Nordic diet also emphasises high-fibre carbohydrate sources (whole-grain barley, oats and rye), berries (e.g. bilberries, lingonberries, cloudberries), fruits (e.g. apples, pears) and vegetables (e.g. cabbages and root vegetables), fatty fish such as salmon, and legumes (e.g. beans and peas). To our knowledge, only few studies on the Nordic diet and depression have been published^(25,26)^. In a cross-sectional study conducted in Norway, Jacka et al. (2011)^(25)^ found an inverse association between *a priori* evaluated diet quality score and elevated depressive symptoms both in women and men. In the *a posteriori* analysis in the same study, the traditional Norwegian dietary pattern was associated with reduced odds of elevated depressive symptoms in men and a healthy dietary pattern with reduced odds in women. Similar evidence was found in the Finnish study, where the prudent dietary pattern, considered a health-promoting diet, was associated with a lower prevalence of depressive symptoms and a lower risk of getting a diagnosis of depressive disorder during the follow-up^(26)^. However, this result was based on an *a posteriori* analysis, where the dietary pattern was created based on the available data rather than using diet quality scores that are based on guidelines of a healthy Nordic diet (*a priori* analysis). The latest case–control study by Araste et al.^(27)^ also reported that high adherence to the Nordic diet was associated with lower odds of depression. Nevertheless, the relationship was found only in the case group of recovered COVID-19 patients.

Because there is very limited research data on the association between adherence to a healthy Nordic diet and the risk of depressive symptoms, we aimed to investigate the association of a healthy Nordic diet score, based on a Baltic Sea Diet Score developed in Finland, with the prevalence of depressive symptoms in middle-aged and older Finnish men.

## Materials and methods

### Study population

The Kuopio Ischaemic Heart Disease Risk Factor Study is an ongoing population-based cohort study designed to investigate risk factors of CVD, other chronic diseases and metabolic conditions in a sample of middle-aged and older men from Eastern Finland. Baseline examinations were carried out in 1984–1989 and 2682 men (82·9 % of those eligible) with ages of 42, 48, 54 or 60 participated. Participants with missing data on baseline diet or depressive symptoms (*n* 79) were excluded, leaving a total of 2603 participants for the analyses. The baseline characteristics of the study population have been described in Salonen et al.^(28)^ All participants gave written informed consent for participation. The protocol of the Kuopio Ischaemic Heart Disease Risk Factor Study was approved by the research ethics Committee of the University of Kuopio, and the dietary part of the Kuopio Ischaemic Heart Disease Risk Factor Study is registered on ClinicalTrials.gov (NCT03221127). This study follows the STROBE guidelines.

### Assessment of food consumption and nutrient intakes

Food and beverage consumption was assessed at baseline with an instructed 4-day food diary with one weekend day. Since the food diary was instructed to be carried out immediately prior to study visits, some food diaries were made over consecutive days and some over nonconsecutive days. To improve the accuracy of the food diaries, participants could use a picture book^(29)^ to support the estimation of portion sizes, and completed food diaries were checked by a registered dietitian together with the participant. Nutrient intakes and the total energy intake were calculated from the food diary using Nutrica® version 2.5 (Social Insurance Institution, Turku, Finland), which used a database built on a Finnish nutrient composition database.

### Diet quality score

In these analyses, a healthy Nordic diet score was used. It is adapted from the Baltic Sea Diet Score, which has been developed to assess healthy diets in Nordic countries^(30)^. Both scores consist of nine components: six food groups (fruits and berries, vegetables, cereals, low-fat milk, fish and meat products) and three nutrients (total fat, the ratio of PUFA to saturated fatty acids and trans-fatty acids and alcohol). A high score is characterised by a better dietary fat quality, a high consumption of berries and fruits, vegetables, whole grains, fish and low-fat dairy and a low consumption of processed meat and alcohol^(31)^. Due to the lack of availability of some dietary components in the Kuopio Ischaemic Heart Disease Risk Factor Study database, there are some differences between the original Baltic Sea Diet Score and the healthy Nordic diet score used in the current study. The main difference is that in the healthy Nordic diet score, whole food groups are used instead of individual foods for some score components (fruits and berries, vegetables and meat products)^(31)^. The description of the components and the differences between the original Baltic Sea diet Score and the healthy Nordic diet score used in the current study is described in Table 1.

**Table 1.** The components and contents of a healthy Nordic diet score

Score component	Description of diet score components of the original Baltic Sea Diet Score	Description of diet score components of a healthy Nordic Diet score	Positive (+)/ Negative score component (-)
Fruits and berries (g/d)	Berries, apples, pears	All fruits and berries	+
Vegetables (g/d)	Tomato, cucumber, cabbage, roots, peas, lettuce	Vegetables, roots, pulses	+
Cereals (g/d)	Rye, oats, barley	Whole grains, excluding rice and pasta	+
Low-fat milk (g/d)	Fat-free milk and milk with < 2 % fat	Fat-free milk and milk with < 2 % fat	+
Fish (g/d)	Salmon, freshwater fish	Salmon, freshwater fish	+
Meat products (g/d)	Beef, pork, processed meat products, sausages	Processed and unprocessed meat	–
Total fat (E%)	Total fat as E%	Total fat as E%	–
Fat ratio	The ratio of PUFA to SFA + trans-fatty acids	The ratio of PUFA to SFA + trans-fatty acids	+
Alcohol	Ethanol	Ethanol	–

E%, percentage of total energy intake.

The diet quality score was derived from food diaries by calculating the mean values of dietary intakes over the four recording days. The healthy Nordic diet score was calculated according to the quartiles of consumption for each score component. Positive score components (fruits and berries, vegetables, cereals, low-fat milk, fish and fat ratio) were given 0–3 points: 0 (zero) for the lowest quartile and 3 (three) for the highest. The points of negative score components (meat products and total fat) were given in an opposite order apart from alcohol, which was given points 0 (ethanol intake ≥ 20 g/d) or 1 (ethanol intake < 20 g/d). The final score (0–25 points) was calculated by summing up the points given for each component. A higher score represents a higher adherence to a healthy Nordic diet^(31)^.

### Assessment of depressive symptoms

Participants completed an 18-item Human Population Laboratory (HPL) depression score questionnaire^(32)^ at baseline. It consists of items dealing with mood disturbance, a negative self-concept, loss of energy, problems with eating and sleeping, trouble with concentration and psychomotor retardation and agitation. The HPL score (range 0–18) was calculated by giving one point for every answer indicating a depressive symptom. The cut-off score of 5 or more indicates a clinically significant depression^(32)^. The HPL depression scale is specially designed for epidemiological studies and is highly correlated with the 21-item Beck Depression Inventory^(32)^. The scale has previously been used to examine, e.g. the relationships between depressive symptoms and serum homocysteine concentrations^(33)^ and dietary folate^(34)^.

### Assessment of other variables

Participants completed self-administered questionnaires related to their marital status, socio-demographic background, history of illnesses, physical activity, smoking and alcohol use at baseline. In this study, marital status is defined as dichotomous (married or living with a partner *v*. other). The variable of adulthood socio-economic status was formed from indicators including current income and the perception of current financial security, current and previous occupations, the highest level of education, housing tenure and the material standard of living^(35)^. A higher score represents lower socio-economic status in adulthood.

The history of CVD and mental illness was recorded with a self-administered questionnaire checked by a study nurse^(36)^. The history of mental illness was assessed with two questions related to previous mental health problems or clinically diagnosed depressive disorder. Positive mental health history was coded if a participant answered ‘yes’ to either (or both) questions. Smoking status (never smoker/previous smoker/current smoker) was determined with questions addressing the frequency and duration of regular smoking, as well as the types of tobacco products used. Participants were classified as current smokers if they had ever smoked regularly and had consumed cigarettes, cigars or pipes within the previous 30 d^(36)^. Alcohol consumption (grams/week) in the preceding 12 months was assessed with a quantity–frequency method using a 15-item Nordic Alcohol Consumption Inventory^(37)^. Leisure-time physical activity was assessed with a 12-month Leisure-Time Physical Activity questionnaire^(38)^, which included the most common leisure-time physical activities of middle-aged Finnish men, as described previously^(39)^. Energy expenditure (kJ/d) was calculated based on the Leisure-Time Physical Activity. Study nurses measured the weight and height of participants, and the BMI was calculated by dividing weight (kg) by height in meters squared.

### Statistical analyses

We used means and linear regression (for continuous variables), the Chi-squared test and the Mantel–Haenszel test (for categorical variables) to assess the univariate associations between the healthy Nordic diet score and the baseline characteristics. The multivariable-adjusted cross-sectional associations between adherence to the healthy Nordic diet and the HPL depression score were analysed with ANCOVA, with the healthy Nordic diet score in quartiles. Using categories, such as quartiles, allows for a more nuanced between-groups comparison without assuming a specific data pattern and also to determine if the effects only present themselves in specific quartiles^(40)^. This approach also mitigates the impact of extreme values from self-reported diet data, thereby enhancing the reliability of the analysis while accommodating variations in diet effects due to biological mechanisms^(40)^. The number of participants is not equal in all quartiles because of the relatively narrow range of the values. Due to the skewness of the HPL score variable, the statistical significance of the differences between the quartiles was assessed using Quade’s non-parametric ANCOVA^(41)^. Sensitivity analysis with complete data was also conducted.

The assumptions of linear regression were not met. Therefore, in addition, a quantile regression analysis was used to analyse the relationship of a healthy Nordic diet score with depressive symptoms using the 0·33 and 0·66 quantiles of the HPL score. Unlike traditional linear regression, which estimates the conditional mean of the response variable, quantile regression estimates the conditional median or other quantiles, such as the 33rd or 66th percentile, of the response variable. Quantile regression is more robust to outliers in the response measurements compared to ordinary least squares regression. It is beneficial when the assumptions of linear regression are violated, such as when residuals are not normally distributed or when there is heteroscedasticity^(42)^. Complete data (*n* 2593) was used in the Quantile regression analysis.

Analyses were adjusted for potential confounders selected a priori based on theoretical relevance and consistent evidence from previous literature^(43–46)^. Descriptive analyses of baseline characteristics across healthy Nordic diet score quartiles were conducted to illustrate population characteristics and contextualise the chosen covariates. Model 1 included examination year, age (years) and daily energy intake (kJ), reflecting the structured baseline assessments conducted between 1984 and 1989 among participants aged 42, 48, 54 or 60 years and accounting for the exclusion of energy intake from the diet score. Model 2 further adjusted for smoking status (never, past, current), leisure-time physical activity (kJ/d), socio-economic status (score) and marital status (married or livings as a couple *v*. other), all of which have been robustly associated with depressive symptoms in prior research^(43–46)^. The covariates were included in the models as continuous variables, except for smoking and marital status, which were treated as categorical variables. Missing values of the continuous covariate ‘leisure-time physical activity’ (*n* 8) were replaced by the mean value of the cohort. With the categorical variable ‘marital status’, missing values (*n* 2) were replaced with the most common answer. *P*-values below 0·05 were considered statistically significant. Data were analysed using SPSS 27.0 for Windows (IBM Corp.) except for quantile regression analysis, performed with SPSS 29.0 R quantile regression extension command^(47)^.

## Results

### Sample characteristics

The average age of the study participants (*n* 2603) was 53·0 years (sd 5·1), with a mean BMI of 26·8 kg/m^2^. The majority of participants were married or living as a couple. More than one-third of participants had a history of CVD, while 5·5 % had a history of mental illness. Nearly one-third of the participants were current smokers. The HPL depression score ranged from 0 to 13 (mean 1·9, sd 2·1), and the healthy Nordic diet score from 2 to 25 (mean 12·8, sd 4·0). Participants with higher adherence to a healthy Nordic diet were physically more active in their leisure time and consumed less alcohol than those with lower adherence to the healthy Nordic diet (Table 2). Participants with a higher healthy Nordic diet score were also more likely to be married or living as a couple, have a higher socio-economic status and be less likely to smoke.

**Table 2.** Sample characteristics according to the quartiles of a healthy Nordic diet score (HNDS), *n* 2603 (Mean values and standard deviations)

Characteristic	HNDS quartiles (score)										
	All *n* 2603		1 (≤ 9) *n* 746		2 (10–12) *n* 487		3 (13–15) *n* 687		4 (≥ 16) *n* 683		*P*-value for trend
	Mean	sd	Mean	sd	Mean	sd	Mean	sd	Mean	sd	
Age (years)^*^	53·0	5·1	52·8	5·0	53·0	5·3	53·0	5·1	53·4	5·2	0·058
BMI (kg/m^2^)^*^	26·8	3·5	26·9	3·6	26·7	3·5	26·9	3·5	26·8	3·5	0·630
Leisure-time physical activity (kJ/d)^*^	594	732	464	690	552	649	632	707	719	824	< 0·001
Energy intake (kJ/d)^*^	10175	2582	10293	2661	10234	2668	10146	2661	10033	2339	0·05
Alcohol intake (g/week)^*^	75	135	93	151	75	105	80	168	50	84	< 0·001
Socio-economic status (points)^*^	9·4	4·6	10·6	4·3	9·4	4·5	9·1	4·7	8·2	4·5	< 0·001
	*n*	%	*n*	%	*n*	%	*n*	%	*n*	%	
Current smoker^†^	825	31·7	351	47·1	159	32·6	184	26·8	131	19·2	< 0·001
Marital status: married or living as a couple^†^	2259	86·9	596	80·1	431	88·5	609	88·6	623	91·2	< 0·001
History of CVD^†^	980	37·6	294	39·4	185	38·0	254	37·0	247	36·2	0·185
History of mental illness^†^	143	5·5	43	5·8	25	5·1	40	5·8	35	5·1	0·722

HNDS, healthy Nordic diet score.

* Linear regression.

† Chi-squared -test and Mantel–Haenszel test.

### A healthy Nordic diet score and depressive symptoms

Lower adherence to the healthy Nordic diet was associated with higher depressive symptom scores (Table 3) when adjusted for examination year, age and daily energy intake (Model 1). The associations remained statistically significant after further adjustments for potential confounders (Model 1 and leisure-time physical activity, smoking, adulthood socio-economic status and marital status). For example, the difference between the highest and the lowest quartile was 0·33 points (95 % CI 0·10, 0·56, *P*= 0·005). In the sensitivity analyses, only men with complete data on all covariates were included (*n* 2593). This had little impact on the associations; for example, the extreme-quartile difference was 0·32 points, 95 % CI 0·09, 0·55, *P*= 0·006 (other data not shown).

**Table 3.** Prevalence of depressive symptoms as assessed with a Human Population Laboratory depression scale in quartiles of a healthy Nordic diet score (HNDS), *n* 2603 (Mean values and 95 % CI)

	Quartile of a healthy Nordic diet score (score)									
	1 (≤ 9) *n* 746		2 (10–12) *n* 487		3 (13–15) *n* 687		4 (≥ 16) *n* 683		*P* for trend	*P* value for differences between the groups^†^
	Mean	95 % CI	Mean	95 % CI	Mean	95 % CI	Mean	95 % CI		
HPL depression score										
Model 1^‡^	2·18	2·03, 2·33	1·94	1·75, 2·12	1·78	1·63, 1·94	1·63	1·47, 1·78	< 0·001	< 0·001
Model 2^§^	2·06	1·90, 2·21	1·93	1·75, 2·12	1·81	1·66, 1·97	1·73	1·57, 1·89	0·003	0·004

HNDS, healthy Nordic diet score; HPL, Human Population Laboratory.

† Assessed with Quade ANCOVA.

‡ Model 1: adjusted for age, examination year and energy intake (kcal/d).

§ Model 2: adjusted for age, examination year, energy intake (kJ/d), leisure-time physical activity (kJ/d), adulthood socio-economic status (points), smoking (never smoker, previous smoker, current smoker) and marital status (married or living as a couple *v*. other).

**Figure 1.**
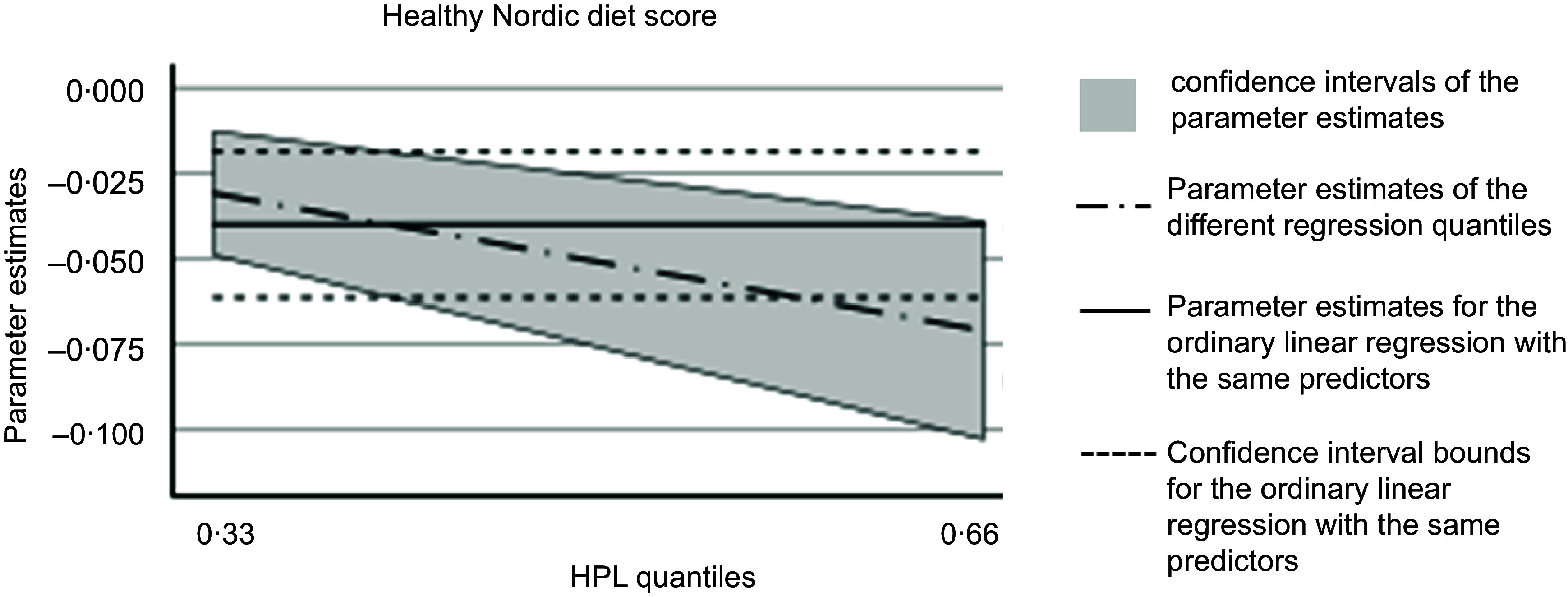
The relationship of a healthy Nordic diet score to depressive symptoms, assessed with the Human Population Laboratory (HPL) depression score, in 0·33 and 0·66 quantiles. The quantile regression analysis was adjusted with age (years), examination year, energy intake (kJ/d), leisure-time physical activity (kJ/d), adulthood socio-economic status (points), smoking (never smoker, previous smoker, current smoker) and marital status (married or living as a couple *v.*. other).

In the quantile regression analysis, a healthy Nordic diet score was associated with the HPL score both in the 0·33 quantile (*β* −0·031; 95 % CI −0·049, −0·013, *P*-value < 0·001) and at the 0·66 quantile of the HPL depressive symptoms score (*β* −0·071 (95 % CI −0·103, −0·039, *P*-value < 0·001), Figure 1.

## Discussion

In this population-based study among middle-aged and older men, we found evidence that higher adherence to a healthy Nordic diet was associated with lower depressive symptom scores after adjusting for potential confounders. The findings of our study are consistent with the previous cross-sectional^(25,26)^ and prospective^(26)^ studies conducted in the Nordic countries. However, neither study defined their dietary pattern as a healthy Nordic diet. In a Norwegian study conducted with 5731 participants, a healthy diet score was associated with 29 % lower odds for depressive symptoms in women and 17 % lower odds in men^(25)^. In addition, a traditional Norwegian dietary pattern was associated with 23 % lower odds for elevated depressive symptoms in men^(25)^. Among women, a healthy dietary pattern was linked to 32 % reduced odds for depressive symptoms^(25)^. Similarly, a Finnish study with 1003 middle-aged men found that a prudent dietary pattern was associated with a 25 % lower prevalence of elevated depressive symptoms^(26)^. In addition, the most recent case–control study conducted in Iran with 240 participants found that higher adherence to the Nordic diet was associated with 24 % lower odds for depressive symptoms in recovered COVID-19 patients^(27)^.

The potential biological mechanisms underlying the association between a healthy diet and depressive symptoms are complex and multifaceted, rather than attributable to a single pathway^(7)^. These mechanisms are influenced by various nutrients, including vitamins, minerals, mono- and polyunsaturated fats, phytochemicals and fibre^(7)^. A healthy Nordic diet, which includes nutrient-rich foods such as berries, fruits, vegetables, whole grains, salmon and freshwater fish, represents this nutrient profile. A recent systematic review of a Nordic diet and its benefits in neurological function suggested that the effect of a Nordic diet on depression might be linked to its modulation of the gut microbiome, reduction of inflammation and mitigation of oxidative stress^(48)^. The high fibre intake from whole grains and vegetables supports a diverse and healthy gut microbiome, which is important for the production of SCFA that have anti-inflammatory properties and support brain health. Furthermore, the rich supply of phytochemicals with antioxidant properties from berries and vegetables helps to mitigate oxidative stress, a contributor to neuroinflammation and depressive symptoms. Moreover, the long-chain *n*-3 fatty acids found in fatty fish like salmon help reduce inflammation by inhibiting the production of pro-inflammatory cytokines^(48)^.

The strengths of this study include the population-based cohort design with a relatively large dataset and the availability of many potential confounding factors. However, there were significant differences across the quartiles of the healthy Nordic diet score in the confounding factors, and the data on these were based on self-report, which reduces accuracy. Therefore, the adjustments may not have fully accounted for the effects of confounding variables. In addition to the study design, food diaries are considered the gold standard method for dietary assessment in population studies, since they allow real-time recording compared to other nutritional assessment methods, such as a FFQ. However, a food diary relies on self-reported information, which is prone to misreporting and may not be representative of the habitual diet or even an accurate diet recording over the assessment period^(49)^. Underreporting or overreporting foods may be influenced by challenges in recalling food intake, difficulties in estimating portion sizes or social desirability, where participants report what they believe is more socially acceptable and considered favourable^(49)^. Also, a 4-day recording of dietary intakes may not accurately represent intakes of foods not consumed frequently. These issues introduce random error to the measurements, which attenuates the associations between dietary factors and outcomes. To improve the accuracy of collected data, picture books of portion sizes were used to assist portion size estimations, and a registered dietitian checked all food diaries. However, food recordings may still contain errors due to lapses in memory, underestimation or random flaws. In addition, since the food diaries were collected only once, the variations in dietary intakes between individual weekdays, weekends or seasons could not be considered. These may increase random error, reducing the possibility of detecting statistically significant associations. Also, the duration of 4 days may not accurately capture foods that are consumed once or twice a week, such as fish, which is known to be linked to a risk of depressive symptoms^(50)^.

The limitations of our study are related to its cross-sectional nature. A cross-sectional study is unable to evaluate changes in populations over time or cause-and-effect relationships. Moreover, a cross-sectional study is unable to determine the direction of the association. Therefore, it might be possible that a higher HPL depression score, indicating a higher level of depressive symptoms, was the reason for a lower-quality diet. In addition, underreporting or underestimation of symptoms has been proven to be common when using self-report depression assessment scales, especially in men^(51)^, which may lead to reporting bias. To our knowledge, no earlier psychometric studies on the HPL depression scale exist, which could give us tools to estimate the direction and magnitude of the possible measurement error. However, in this study, the prevalence of depressive symptoms was 10·9 %, which is similar to the prevalence (12·5 %) previously reported in Finnish men by Saltevo et al.^(52)^

Since this study focused on middle-aged or older males only, the results may not be generalised to younger male generations or women. Furthermore, depression is known to be a gendered phenomenon; it is more common among women than men^(52,53)^. This difference emerges already during puberty and remains stable in adulthood^(54)^. Additionally, the depressive disorder also manifests differently in men compared with women^(54)^. For instance, men are more likely to show symptoms of irritability, aggression, substance abuse and increased risk behaviour^(55,56)^. In contrast, women are more likely to experience increased appetite, hypersomnia and somatic and cognitive-affective symptoms^(55,57–60)^, and their symptoms are more severe^(56)^. Moreover, conventional depression screening instruments might not be sensitive enough to screen male-specific symptoms such as irritation or aggression^(60)^. Consequently, these gender differences in symptomatology may lead to the under-recognition of depressive symptoms and depressive disorder in men. Furthermore, the diet quality score used in this study to define a healthy Nordic diet might have affected the findings, as the score was adapted from the Baltic Sea Diet Score with modifications due to data availability on certain foods. Although both scores aim to capture the typical Nordic diet, the differences in their included components may affect how much the score reflects a healthy Nordic diet and its effectiveness in predicting disease risk.

In conclusion, this cross-sectional study shows that higher adherence to the healthy Nordic diet is associated with lower depressive symptom scores. Further research using prospective designs and randomised controlled prevention trials is required to investigate the role of the healthy Nordic diet in the risk of the onset of depressive symptoms.
